# Prenatal Alcohol Exposure Inhibits Transient Expression of Autophagy and Synaptic Proteins in Developing Brain

**DOI:** 10.26502/ogr0173

**Published:** 2025-01-22

**Authors:** Monica Hampe, Nune Darbinian, Nana Merabova, Armine Darbinyan, Jamal Hamze, Uday Bharai, Yuri Persidsky, Mary F Morrison, Shohreh Amini, Laura Goetzl, Michael E Selzer

**Affiliations:** 1 Center for Neural Development and Repair, Department of Neural Sciences, Lewis Katz School of Medicine at Temple University, Philadelphia, PA 19140, USA.; 2 Department of Obstetrics and Gynecology, Gundersen Health System, La Crosse, WI 54601, USA.; 3 Department of Pathology, Yale University School of Medicine, New Haven, CT 06520, USA.; 4 Department of Pathology, Temple University, Lewis Katz School of Medicine at Temple University, Philadelphia, PA 19140, USA.; 5 Department of Psychiatry, Lewis Katz School of Medicine at Temple University, Philadelphia, PA 19140, USA; 6 Department of Biology, College of Science and Technology, Temple University, Philadelphia, PA 19122, USA.; 7 Department of Obstetrics, Gynecology and Reproductive Sciences, McGovern Medical School at The University of Texas Health Science Center at Houston (UTHealth), Houston, TX 77030, USA.

**Keywords:** Alcohol, FASD, Autophagy, Fetal Brain, Synaptic Plasticity, Apoptosis, Exosomes

## Abstract

**Introduction:**

Neuronal apoptosis and consequent inhibition of autophagy, with loss of synaptic connections are central events in the genesis of fetal alcohol spectrum disorders (FASD). However, studies of molecular mechanisms of autophagy in human fetal brain are limited. Recently, prenatal exposure to EtOH was associated with reduced miRNA-9 levels in fetal brain-derived exosomes (FB- Es) isolated from maternal plasma, which correlated with small eyes, an anatomical hallmark of fetal alcohol syndrome (FAS). Since miR-9 targets several genes that regulate synaptic plasticity, EtOH-induced inhibition of miR-9 could potentially result in dysregulation of genes involved in synaptogenesis/plasticity.

**Methods:**

Rats were fed a 6.7% EtOH liquid diet from E16 to birth. Human studies: Fetal brain tissues from elective pregnancy terminations were collected at 9–23 weeks gestational age (GA; n=20 EtOH-exposed and 20 GA- and fetal sex-matched unexposed controls). EtOH consumption was assessed by questionnaire (adapted from NIAAA PASS). Expressions of 84 genes in a synaptic plasticity microarray were assessed in human fetal brain samples, verified by qRT-PCR, and for some mRNAs, copy number was determined in FB-Es by droplet digital PCR. Protein expression was measured in brain by qWestern blot assays or with a MAP kinase microarray. Exosomal protein levels were measured by ELISA.

**Results:**

Levels of pro-apoptotic caspase-3 and Bax were significantly increased in the brains of EtOH-exposed rat pups, while early expressions of anti-apoptotic Bcl2 and biphasic Bag3 were inhibited. Phosphorylation of GSK3β was increased, and during Bag3 inhibition, the GSK3β target β-catenin also was increased. EtOH-exposed P8 and P15 rats showed motor abnormalities during low Bag3 expression. EtOH exposure reduced expression of synaptophysin and synapsin. In most synaptic plasticity pathways, levels of mRNAs were reduced. Several immediate-early genes were upregulated, but SYNPO, which is involved late in synaptic plasticity was downregulated 78%. Genes involved in Long Term Potentiation (LTP) and long-term depression (LTD) were downregulated, but the neurotoxic cytokine TNF⍺ was upregulated.

**Conclusions:**

Prenatal exposure to EtOH was associated with reduced expression of autophagy genes in the fetal brains of rats and humans. Inhibition of Bag3 correlated with upregulation of GSK3β and its downstream targets, suggesting dysregulation of β-catenin signaling. Synaptic proteins, including those implicated in LTP and LTD also were inhibited by EtOH. The results in FB-E mirrored those in brain tissue. Reduced expression of miR-9 target synaptic genes in FB-Es might serve as novel biomarkers to predict FASD.

## 1. Introduction

Fetal alcohol spectrum disorders (FASD), including the most severe form, fetal alcohol syndrome (FAS), are characterized by significant neuronal and axonal loss, developmental delay, and multiple somatic disorders, including stereotypical facial abnormalities [1, 2]. In the United States, the incidence of FASD is estimated at nearly one in every 100 live births. In pregnant women, rates of ongoing EtOH use and binge drinking are at least 7.6% [3]. Despite recent research on the mechanisms of neuronal injury in the EtOH-exposed human fetal brain, significant gaps in knowledge remain.

### EtOH mediated neurotoxicity: key pathways.

Effects of EtOH on the developing CNS include reduced neurogenesis, and induction of apoptosis [4]. In animal models, the pattern of injury depends on the developmental stage at exposure. A single dose (35–42 mg/dl) of EtOH at gastrulation is associated with decreased neural crest cell numbers but does not affect neural crest cell migration patterns [5]. In primates, early and late gestational exposures to EtOH were said to be equally detrimental to neonatal behavior [6]. EtOH exposure during the 2nd trimester may alter the fate of neural stem and progenitor cells in vitro [7]. Key mechanisms underlying EtOH-associated damage of the developing brain include activation of caspase-3-mediated apoptosis, inhibition of synaptic plasticity and serotonin-dependent plasticity [8, 9]. In human fetal cells, EtOH exposure was associated with increased susceptibility to oxidative stress and cell damage, activation of apoptosis, and reduced expression of genes associated with neuronal plasticity [24]. The important role of the activation of oxidative stress pathways in causing EtOH-induced neuronal cell injury was also shown in rats in vivo [10]. Microarray gene expression profiling in animal models have identified EtOH-related alterations in cellular processes/pathways including genes involved in axon guidance and synaptosome formation [11, 12]. However, these findings have not been validated in human fetal development in vivo. The toxic effects of alcohol exposure on fetal brain development have been discussed extensively, and several biomarkers for early detection of FASD have been proposed [1, 13, 14, 15]. Prenatal exposure to alcohol also was associated with downregulation of miRNAs in fetal brain [16]. This was accompanied with significant reductions in expression of several synapse-related genes (SYNPO, Synapsin), but with increased expression of several cytokines, including the neurotoxic TNF-α. Other studies provided evidence that alcohol exposure may inhibit autophagy, while simultaneously increasing protein levels of caspase-3 in human brain [17]. However, not much is known about the role of the autophagy pathway, or its alterations due to maternal alcohol consumption, in the pathogenesis of FASD. Although, we previously demonstrated the effects of prenatal EtOH exposure on expression of fetal brain miRNAs [16], effects on the miRNA-targeted synaptic plasticity pathway have not yet been explored.

### EtOH mediated inhibition of autophagy.

EtOH exposure can inhibit autophagy, a cellular mechanism that acts as a protective response for the CNS that is involved in cell survival, metabolism and immunity. In experimental animals, Bcl2-associated athanogene-3 (Bag3) is implicated in selective autophagy and plays an important role in the clearance of misfolded proteins in the developing brain. Its inhibition by EtOH contributes to excessive apoptosis [17]. Activation of the autophagic pathway also increases the levels of LC3-II complexes in the liver, indicating the formation of functional autophagosomes, while pharmacological or genetic inhibition of autophagy increases intracellular p62/SQSTM1 levels, a degradation product of autophagy [17]. Thus, chronic alcohol consumption significantly impairs autophagic mechanisms in the liver, but less is known about the impact on fetal brain, and even less in human fetal brain.

### Fetal brain-derived exosomes.

To evaluate the effects of EtOH on fetal development, we recently studied the expressions and activities of neuronal and OL markers and miRNAs in fetal brain-derived exosomes (FB-E) during pregnancy [13, 14, 15, 16]. FB-E can cross the placenta and enter the maternal blood, from which they can be isolated non-invasively from maternal blood [14, 18]. In the present study, we focused primarily on defining the alcohol exposure-associated changes in expression of synaptic plasticity and autophagy gene and protein pathways in FB-E.

## 2. Results

Recently, we described EtOH-associated neuronal injury in vitro and in an in vivo rat FAS model [19, 10]. We also suggested a possible mechanism for EtOH neurotoxicity caused by dysregulation of miRNAs [16]. Here, we examine the effect of EtOH exposure on synaptic plasticity genes, which are downstream targets of miRNAs, in a rat model of FASD, in human fetal brain, and in human FB-E.

### 2.1. EtOH exposure is associated with a decrease in cell viability in neuronal cells.

**FIGURE 1:**
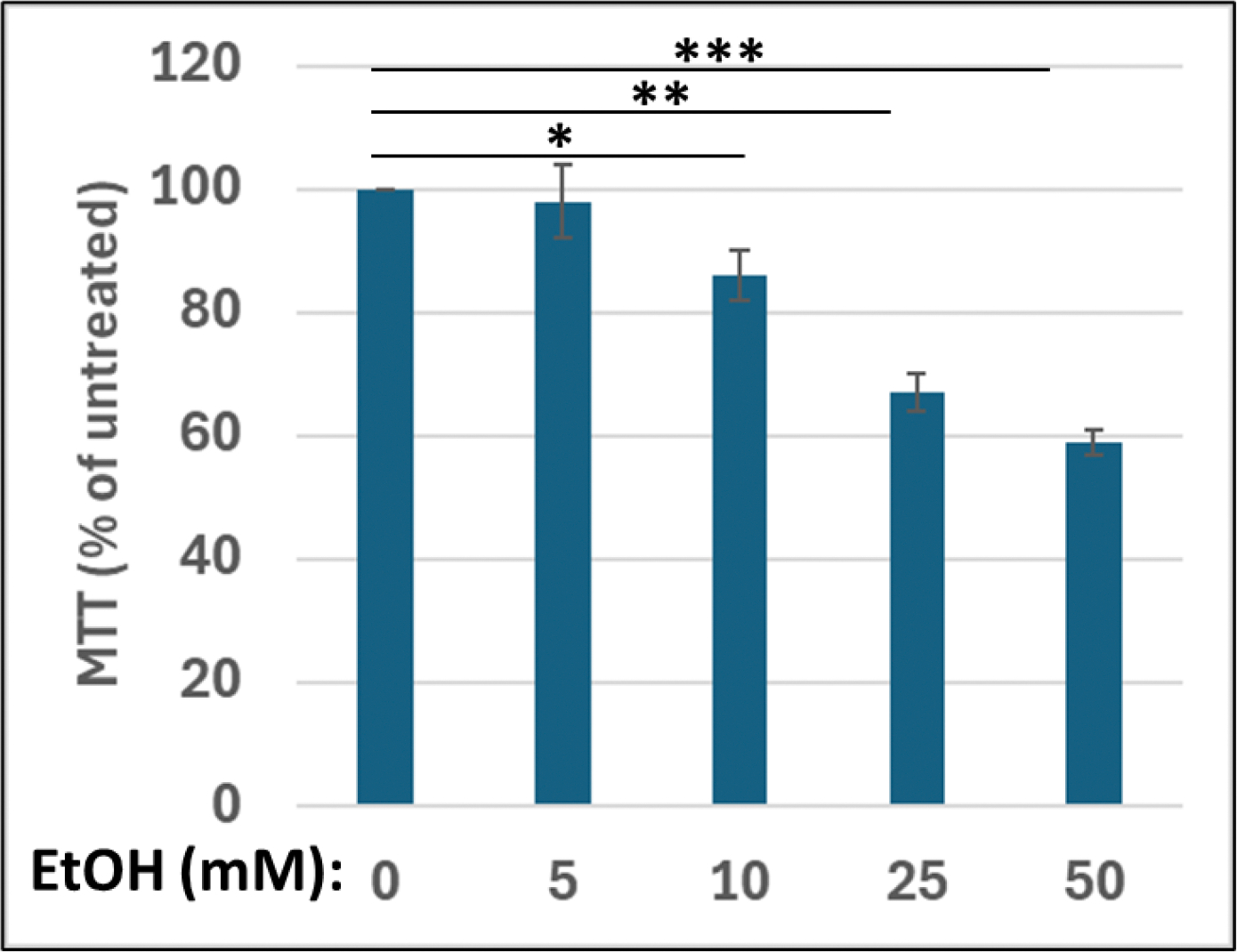
In vitro human FASD model: EtOH-induced neuronal cell injury. Cell metabolism/activity (MTT) assay in human cortical neurons incubated with increasing concentrations of ethanol (from 5 to 50 mM). Equal numbers of cells were plated in triplicate, and then treated with EtOH. MTT assay illustrating neuronal cell metabolism and activity in the presence of EtOH. The values are derived from three independent experiments. Cell viability determined by methylthiazole tetrazolium (MTT) assay was quantified by spectrophotometry at 570 nm with a reference wavelength of 650 nm. The numbers represent the average of three independent experiments. Bar 1 represents untreated cells set as 100%.

Using cell metabolism/activity (MTT) assay, we demonstrated a dose-dependent decline (up to 40%) in metabolic activity in the presence of EtOH compared with controls (Figure 1) in human cortical neurons incubated with increasing concentrations of EtOH from 5 to 50 mM.

### 2.2. EtOH exposure differentially affects the autophagy stress-inducible anti-apoptotic Bcl2-associated athanogene-3 (Bag3) protein and MAPK substrates in brain.

**Figure 2.**
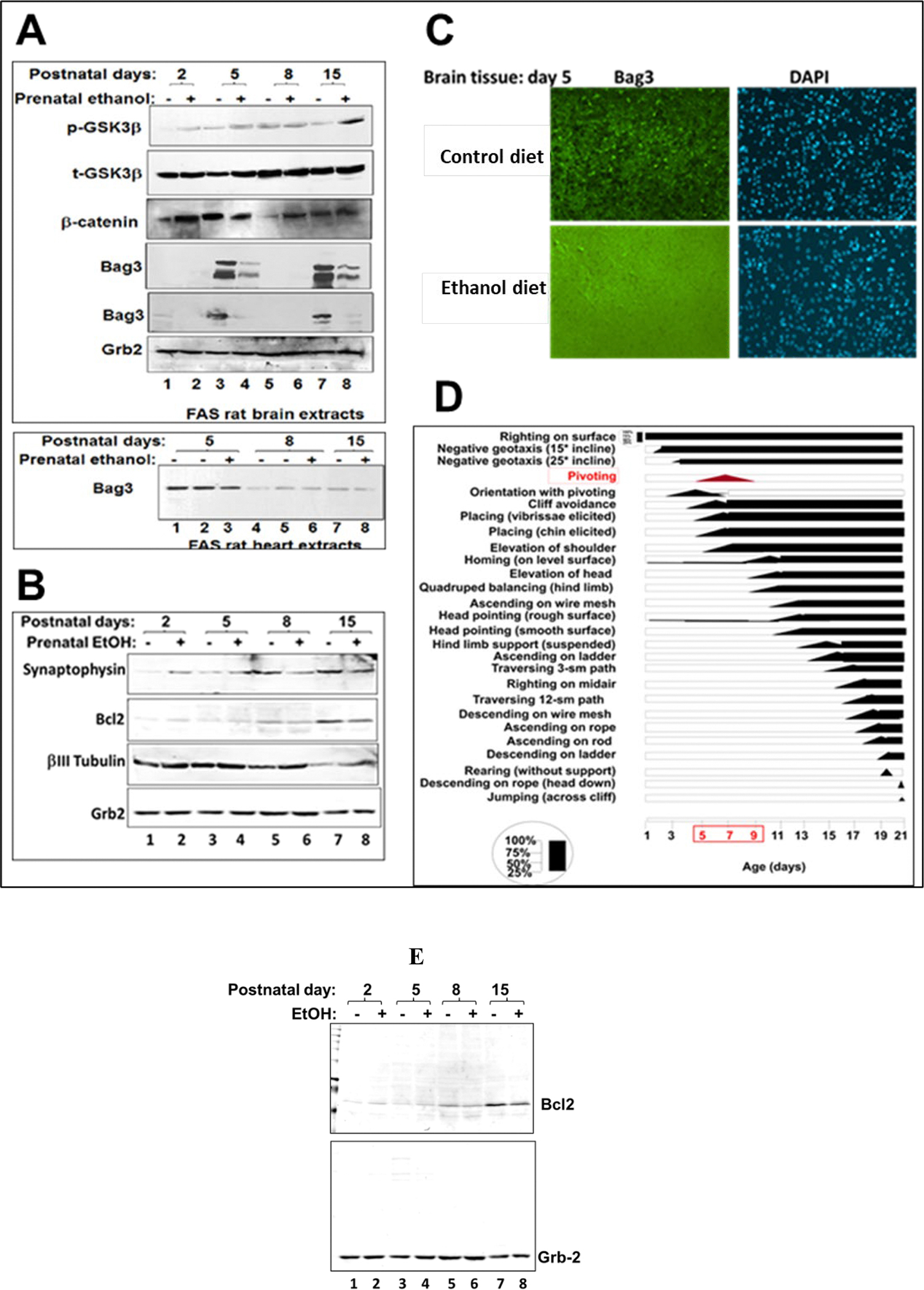
Differential effects of EtOH exposure and gestational age on MAPK substrates and autophagy stress-inducible protein Bag3 in brain, but not in heart tissues in rat FASD model. **A.** Western blot analysis for developmental expression of total and phosphorylated GSK3β, β-Catenin and Bag3 in brain tissues from control or EtOH diet groups at postnatal days P2 to P15. Early expression of the anti-apoptotic signal Bag3 was inhibited by EtOH exposure. Equal loading was verified by using anti-Grb2 antibody. The upper panel of Figure 2A shows the effect of EtOH on brain Bag3 levels. The upper Western blot shows the response to EtOH (high intensity image) and the lower image is a low intensity image. The Bag3 level fluctuation was specific for brain and was only slightly decreased in heart tissues (Figure 2A, bottom panel). **B.** Effect of EtOH on fetal synaptic proteins. Western blots analysis using rat brain protein extracts shows developmental regulation of Synaptophysin, increase in level of expression at postnatal P15, and negative effect of ethanol diet on Synaptophysin expression at postnatal days P2 to P15. **C.** Levels of Bag3 were studied at postnatal day P5 by immunohistostaining. Decrease in Bag3, and slightly decrease in cell number (DAPI nuclear staining) were confirmed in EtOH diet group (bottom panels). DAPI staining in brain tissues at postnatal P5 demonstrating a smaller number of cells in EtOH diet group compared to control diet. **D.** Behavioral observations at postnatal stages of brain development from P5 to P8: Bag3 fluctuation period is important for pivoting. Summary diagram of the different locomotor, and other related skills in rats. In the majority of instances performances levels (25, 50, 75, and 100 percent) refers to the percentage of rats successful in the display of the response frequency. Adapted and redrawn from Altman and Sudarshan (1992). **E.** Image of the whole membrane on developmental expression of Bcl2 shown in western-blot assay. Grb2 was used as a loading control. Lower levels of pro-survival Bcl2 were found in brain of Ethanol diet groups at later postnatal days, P8 and P15.

EtOH exposure increased caspase-3 activity in neonatal rat brain [10], which is equivalent to the late term fetus in humans. Here, we studied effects of EtOH on key signaling proteins in rat brain (Figure 2). Developmental expression of total and phosphorylated GSK3β, β-Catenin and Bag3 was studied in brain tissues from control or EtOH diet groups at postnatal days P2 to P15 (Figure 2A). The early expression of anti-apoptotic Bag3 was inhibited by ethanol exposure (Figure 2A, lanes 2, 4, 6, 8). Bag3 levels were verified in independent western-blot assays (panels 4 and 5). Higher exposures for Bag3 are shown in panel 4. Bag3 level fluctuation was specific for brain and was only slightly decreased in heart tissues (bottom panel). Next, we demonstrated EtOH-associated decrease of synaptic protein, Synaptophysin, at postnatal days P8 and P15 (Figure 2B, lanes 6, 8), while there was developmental increase in the level of expression of Synaptophysin at postnatal days from P2 to P15 (Figure 2B, lanes 1, 3, 5, 7). Meanwhile, anti-apoptotic Bcl2 was also decreased in EtOH group at postnatal days P8 and P15. Bag3 level changes were studied at postnatal day P5 by immunohistostaining, and the decrease in Bag3 level was confirmed in EtOH diet group (Figure 2C, bottom panels). Behavioral observations at postnatal stages of brain development from P5 to P8 were performed and demonstrated that Bag3 fluctuation period was correlated for pivoting (Figure 2D). Summary diagram of the different locomotor, and other related skills in rats is shown in (Figure 2D). The performance level (25, 50, 75, and 100 percent) refers to the percentage of rats successful in the display of the response frequency. Diagram was adapted and redrawn from Altman and Sudarshan, 1987; Bayer and Altman, 1992 [20, 21]. Finally, the image of the whole membrane for the developmental expression of Bcl2 was shown (Figure 2E) to confirm the purity of the bands. Lower levels of pro-survival Bcl2 were found in brain of Ethanol diet groups at later postnatal days, P8 and P15. These observations suggest that EtOH-induced autophagy inhibition may be a primary cause of the activation of apoptotic pathways. Next, we looked at apoptotic caspase-3 in human fetal brain synaptosomes.

### 2.3. Prenatal EtOH exposure is associated with increased caspase-3 activity in human fetal brain synaptosomes during 1^st^ and 2^nd^ trimester.

**Figure 3:**
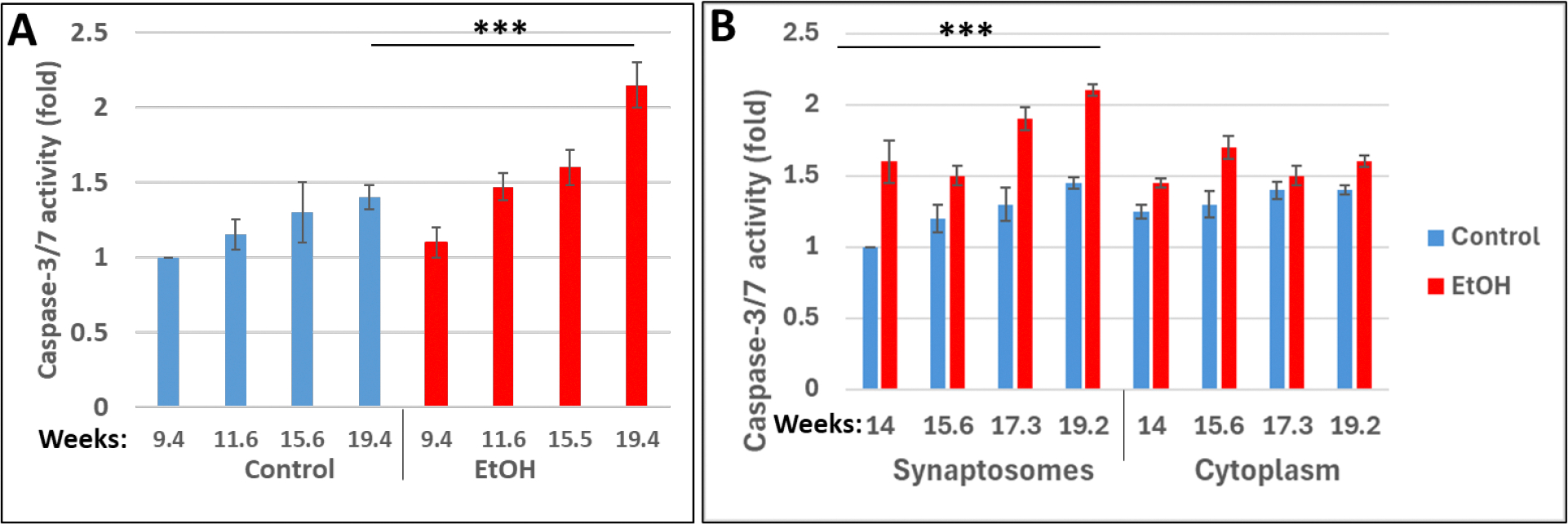
Prenatal EtOH exposure is associated with increased caspase-3 activity in human fetal brain synaptosomes during 1^st^ and 2^nd^ trimesters. **A.** Toxic effects of alcohol on caspase-3 in synaptosomes isolated from human fetal brains, exposed to EtOH. Fetal synaptosomes were analyzed by GLO-assay for activation of Caspase-3 in fetal brain synaptosomes. **B.** EtOH exposure and gestational age: effect on fetal synaptosome caspase-3 activation. Synaptic and cytoplasmic extracts were prepared using Syn-Per Synaptic Protein Extraction Reagent (Thermo Scientific). Caspase-3/7 cleavage was measured in fetal brain tissues by GLO caspase-3/7 apoptotic assay. Apoptosis was assessed by analysis of activation of Caspase-3 using the substrate DEVD- amino luciferin from Caspase-Glo^™^ 3/7 assay kit (Promega, Madison, WI, USA). Luminescence was recorded as RLU/sec on a Turner Designs Luminometer TD-20/20 (Promega). The values represent the results from these experiments. Caspase-3 is increased with EtOH exposure in brain at all developmental stages compared to controls.

Toxic effects of alcohol on caspase-3 in synaptosomes isolated from human fetal brains, exposed to EtOH. Fetal synaptosomes were analyzed by GLO-assay for activation of Caspase-3 in fetal brain synaptosomes. EtOH exposure and gestational age: effect on fetal synaptosome caspase-3 activation. Synaptic and cytoplasmic extracts were studied for Caspase-3 activity, which was increased with EtOH exposure in brain at all developmental stages compared to controls (Figure 3). We developed a human biobank (with prenatal exposure to ethanol), where we confirmed toxic effects of ethanol on brain cells, and continued studies in brain synaptosomes. Clinical characteristics of subjects used in this study are presented in Table 1. Maternal blood and fetal brain samples from EtOH-exposed cases (n=20) and Controls (n=20) were matched individually for fetal sex, GA, and maternal age. PCR for SRY gene on Y chromosome for sex determination was performed for EtOH cases and control samples. Here, we have quantified the adverse effects of EtOH exposure on the marker of apoptosis, active Caspase-3. We isolated synaptic vesicles from four fetal brain samples from women and measured Caspase-3 activity using the GLO 3/7 assay (Figure 3A) as described in our prior publications [23, 24, 25] and demonstrated increased Caspase-3 activity during 2nd trimester. Our data also demonstrate that Caspase-3 levels are significantly higher in the cytoplasm (p<0.001) compared to controls, and trended higher in the syntaptosomes (p<0.0011) isolated from EtOH exposed human fetal brain tissue (Figure 3B). Levels of Caspase-3 in synaptic membranes and cytoplasm were similar (p=0.57). Synaptic Caspase-3 levels increased with increasing gestational age (r=0.73, p=0.04). It is possible that an increasing apoptosis is due to gestational age associated susceptibility to EtOH toxicity, or it could be from the cumulative effects of EtOH with longer exposure. Our data suggest that regulated apoptosis is a critical neurodevelopmental event, since a degree of increasing apoptosis is seen in controls, but the increased apoptosis may be critical. Thus, we observed a positive correlation between Caspase-3 activation and increasing gestational age suggesting that some activation of Caspase-3 is part of normal development. Caspase-3 is increased with EtOH exposure in both the cytoplasm and the synaptic membranes compared to controls. Apoptosis plays an important role in plasticity of developing neuronal connections. However, Caspase-3 activation was enhanced with EtOH exposure, and these changes can lead to further abnormal fetal development. Thus, our results showed that activation of Caspase-3 was markedly higher in ethanol groups compared to controls at all developmental time points.

### 2.4. Exposure to EtOH is associated with reduced expression of genes involved in synaptic plasticity in fetal brain.

**Figure 4:**
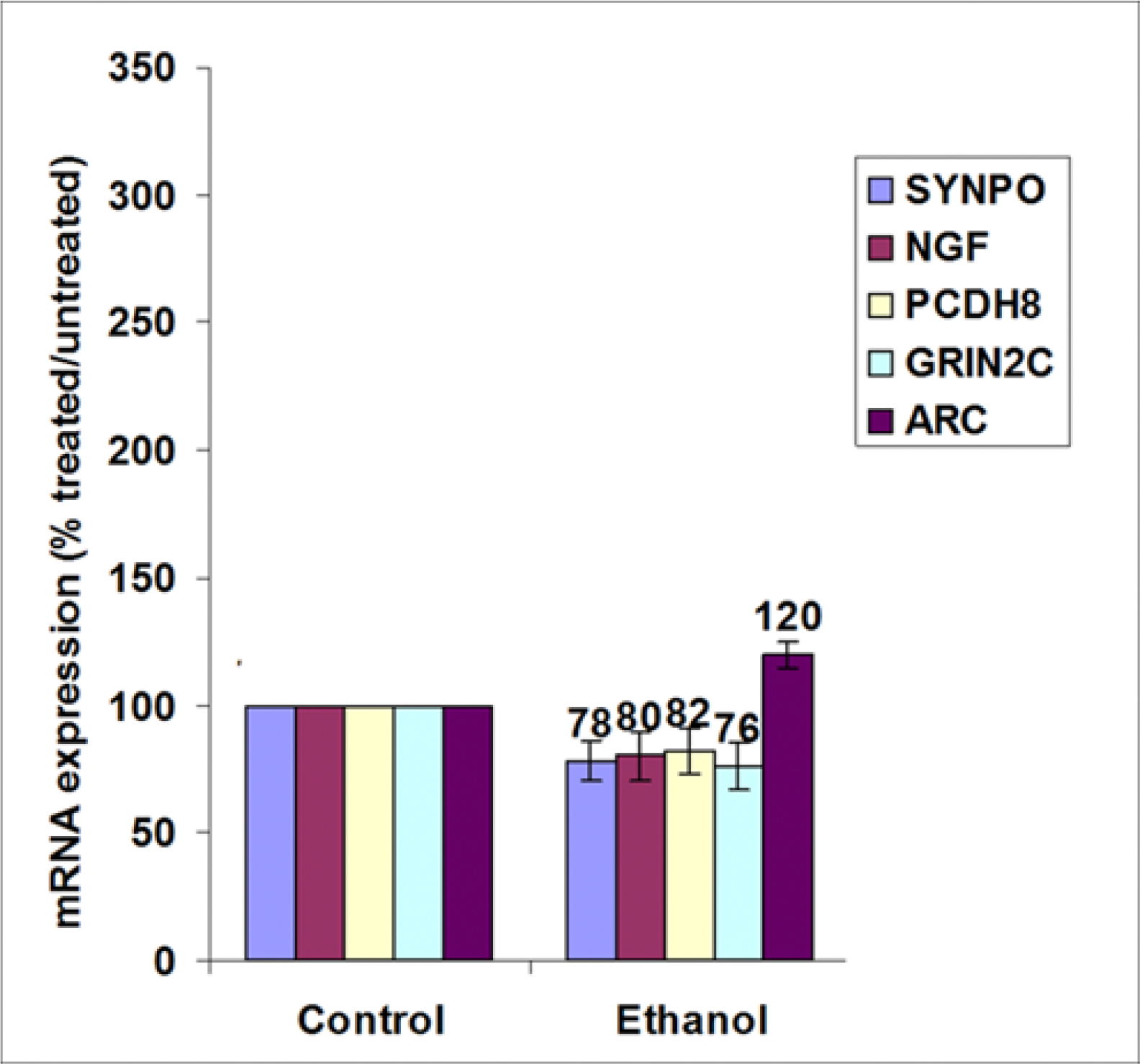
Exposure to EtOH is associated with reduced expression of genes involved in synaptic plasticity in fetal brain. Synaptic plasticity array was performed using RNAs isolated from human fetal brain.

Synaptic plasticity array was performed using RNAs isolated from human fetal brain. EtOH-induced neuronal injury can be in part mediated via regulation of synaptic gene expression. Brain lysates were analyzed for genes involved in synaptogenesis pathways (Table 2) in particular, i) immediate-early response genes (IEGs): NGF, nerve growth factor [beta polypeptide], ii) immediate-early response genes (IEGs) / cell adhesion: PCDH8, Protocadherin 8, iii) late response genes: SYNPO, Synaptopodin, iv) postsynaptic density / late response genes: ARC, Activity-regulated cytoskeleton-associated protein, v) long term potentiation (LTP) / CREB cofactors / neuronal receptor: GRIN2C, Glutamate receptor, ionotropic, N-methyl D-aspartate 2C (NMDAR2C, NR2C). We show that EtOH exposure is associated with downregulation of synaptic genes (Figure 4). Synaptophysin (SYNPO), NGF, PCDH8 and GRIN2C expression was inhibited in EtOH group, while ARC gene was slightly upregulated. (Figure 4). Major human synaptic plasticity genes implicated in CNS development and disease were assayed in this study and presented in Table 2.

### 2.5. Prenatal exposure to EtOH is associated with increased phosphorylation of p-JNK2 (MAPK signaling) in fetal brain.

**Figure 5:**
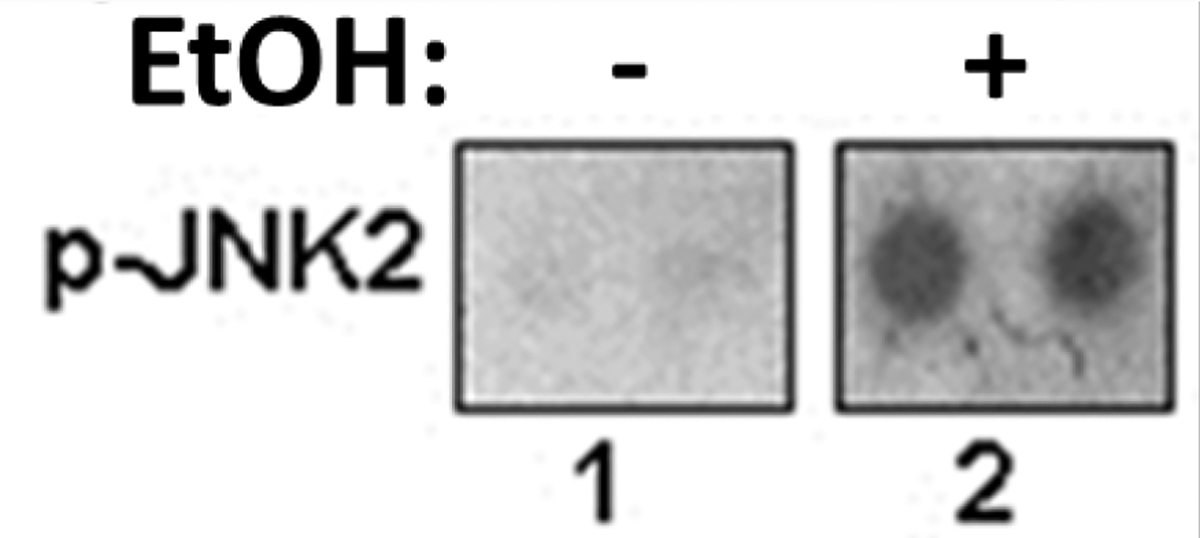
Prenatal exposure to EtOH is associated with increased p-JNK2 phosphorylation (MAPK signaling) in fetal brain. EtOH induced serine/threonine phosphorylation hyperphosphorylation of JNK2. Phospho-MAPK array was performed using protein lysates from fetal brain tissues. As indicated, EtOH increases serine/threonine phosphorylation of key MAPK signaling molecule, JNK2.

Phosphorylation of key proteins involved in signal transduction pathways, including MAPK signaling represents an important posttranslational modification. Thus, next we looked at MAPK/ERK1/2 cascade that modulates expression of GSK3β controlling neuronal progenitor proliferation and establishment of neuronal polarity during development, and throughout the lifespan. It was also shown that in CNS neurons, c-Jun activity in stress-induced apoptosis is regulated by its N-terminal phosphorylation. Phosphorylation of c-Jun in CNS neurons can be regulated by Jun N-terminal kinases, JNKs. Here we demonstrate that ethanol induces phosphorylation of c-Jun kinase, JNK2, in fetal brain (Figure 5). Effect of EtOH on serine/threonine phosphorylation of MAPK signaling in brain cells is shown in Figure 5. Results from phospho-MAPK array indicated that EtOH increases serine/threonine phosphorylation of key MAPK signaling molecule, JNK2. EtOH induced serine/threonine hyperphosphorylation of JNK2. Phospho-MAPK array was performed using protein lysates from fetal brain tissues. As indicated, EtOH increases serine/threonine phosphorylation of key MAPK signaling molecule, JNK2. We compared the effects of EtOH exposure on phosphorylated status of MAPK kinases. We showed that prenatal EtOH exposure was associated with an 8-fold increase of p-JNK2 phosphorylation (Figure 5), compared to unexposed controls. Thus, induction of serine/threonine phosphorylation of MAPK pathway by EtOH could be one of mechanisms of activation of apoptotic events in fetal brain.

### 2.6. Differential effects of EtOH on the expression of synaptic markers.

EtOH exposure is associated with a decrease in expression of majority of synaptic plasticity genes and autophagy proteins, and with an increase of apoptotic proteins in murine and human fetal brain and neuronal cells, and in human FB-Es. In the present study, we compared maternal blood and fetal brain tissues from 20 EtOH-exposed cases from 1^st^ (9 to 14 weeks) and 2^nd^ (14.1 to 23 weeks) trimester pregnancies with 20 GA-matched controls. The clinical characteristics of the subjects are presented in Table 1. The levels of mRNA or protein markers of synaptic plasticity, apoptosis and autophagy, were assayed in whole brain homogenates and FB-Es by qRT-PCR, ddPCR, ELISA and western-blot assay, and presented as fold regulation in Table 3. Values are normalized relative to controls (n = 20 per group, GA=9 to 23 weeks for Control and EtOH-exposed group). We demonstrated that EtOH exposure increases expression of cytotoxic cytokines/chemokines and apoptotic proteins in fetal brain and inhibits survival and autophagy proteins. Cytokine transcription was assayed by Real-Time qRT-PCR for TNF-α and IGF-1 mRNA in human fetal and rat pups brain. In animal studies n = 16 pups total, by 4 pups per each age group, in the EtOH-exposed group, and control groups. In rat brain: Caspase-3 and Bax were increased in the brain of EtOH pups (↑1.9 and ↑2.3), while the early expression of anti-apoptotic Bcl2 and biphasic Bag3 was inhibited by EtOH (1.7 and 8.2 folds). Phosphorylation of GSK3β was increased with EtOH exposure. During the period of Bag3 inhibition, the level of GSK3β downstream target, β-catenin, was also increased in the brain of EtOH pups. Behavioral observations at postnatal days P8 and P15 revealed pivoting difficulties in the ethanol group when compared to controls, coinciding with low Bag3 expression, and reduced synaptophysin. In human FB-Es: the significantly reduced expression of synaptic markers, synaptophysin, synaptotagmin, synaptopodin, neurogranin and synapsin were found in EtOH group, compared to controls, in the fetal brain and FB-Es. Decreased levels of neuronal survival factors were found in EtOH group. The significant differences were between levels of HSF1, Bcl-XL, and REST for the control and EtOH groups. Up to 5-fold inhibition in microRNA-9 was observed in FB-Es from EtOH exposed groups compared with controls. In human fetal brain: in most synaptic plasticity pathways, levels of mRNAs were reduced (but ARC, MMP9 and TNF). Immediate-Early Response Genes (IEGs): ARC (↑120%), ↓BDNF, ↓CEBPB, ↓JUN, ↓JUNB, ↑MMP9, ↓NFKB1, ↓NGF (80%), ↓PCDH8 (82%), ↑TNF. Late Response in Synaptic Plasticity: ↓SYNPO (78%). Genes involved in Long Term Potentiation (LTP): ↓BDNF, ↓GRIN2C (76%), ↓MAPK1 (ERK2); Long Term Depression (LTD): ↓IGF1, ↓MAPK1 (ERK2); Cell Adhesion Molecules: ↓PCDH8, ↑TNF; Extracellular Matrix (ECM) Molecules: ↑MMP9; CREB Cofactors: ↓AKT1, ↓GRIN2C (76%), ↓MAPK1 (ERK2); Neuronal Receptors: ↓GRIN2C (78%); Other Synaptic Plasticity Genes: ↓SIRT1. Upregulated in EtOH cases, including the neurotoxic TNF⍺, immediate early gene ARC, which is trafficked to dendrites, and MMP9.

## 3. Discussion

Massive apoptosis of neurons by activation of Caspase-3, resulting from exposure of the developing brain to alcohol, is considered as the main cause of long-lasting structural CNS abnormalities and neuronal cell death. GSK3β and its downstream target β-catenin play an essential role in neural development. Modulation of GSK3β in brain is a major focus for treating neuropsychiatric and neurodegenerative diseases. Previously, we established an in vivo rat FAS model [10], and demonstrated that the level of pro-apoptotic Caspase-3 was increased in the brain of pups in the EtOH group. Here, we show that the early expression of anti-apoptotic Bag3 was inhibited by EtOH exposure in the same pups’ brains. Bag3 plays an important role in protein quality control and the clearance of misfolded proteins in the developing brain. Fluctuating between high (postnatal day P5 and P15) or low levels of Bag3 at early stages (P2 and P8) is probably due to some developmental processes. Probably pivotal stage in development is important stage when Bag3 should not be present at that time and its expression should be silent during that period (P8). The level of GSK3β playing an essential role in neural development was increased in relation to the decreased Bag3 in the ethanol-treated groups. During the period of Bag3 inhibition, the level of a GSK3β downstream target, β-catenin, was also increased in the brain of ethanol-exposed pups, suggesting its accumulation in cells and dysregulation of β-catenin signaling that can possibly induce developmental brain defects.

### 3.1. Autophagy and synaptogenesis in FASD.

Developmental expression of autophagy-specific Bag3, that plays an important role in protein quality control and in the clearance of misfolded proteins in the developing brain, was inhibited by ethanol exposure. Bag3 level fluctuation was specific for brain only and was only slightly decreased in heart tissues. Bag3, which stabilizes the level of anti-apoptotic Bcl-2 family, is a chaperone targeting misfolded proteins. While EtOH activates the expression of pro-apoptotic Bax and Caspase-3, it inhibits anti-apoptotic Bcl2 protein. The newly synthesized proteins are targeted to the activated synapses. One of synaptic proteins, Synaptophysin was also strongly inhibited by ethanol. Developmental expression of Synaptophysin was increased from P2 to P15 but was negatively affected by EtOH. In contrary, βIII Tubulin was negatively regulated upon development in agreement with published literature. It was previously shown for βIII Tubulin to be inhibited at transcription level during normal developmental processes. Studies on beta III tubulin, demonstrated that its expression in control samples decreased, as expected, upon brain development and neuronal outgrowth and axon elongation. Behavioral observations (adapted from Altman and Sudarshan (1992)) revealed that Bag3 fluctuation period at postnatal stages of brain development from P5 to P8 is important for pivoting, as summary diagram of the different locomotor, and other related skills in rats is shown in Figure 2D. Thus, prenatal exposure to ethanol affected the early postnatal expression of Bag3 and apoptosis-related proteins and other developmentally regulated proteins in the brain, including synaptophysin. Hence, here, we demonstrate for the first time that while bi-phased Bag3 expression is low in developing brain and accumulates with aging, it is also strongly affected by prenatal ethanol exposure that leads to the dramatic changes in key metabolic pathways in brain. Our previous animal studies demonstrated also a negative correlation between prenatal ethanol uptake and the development of the brain [10]. Body and brain weight were significantly lower at P5 and P8 in the EtOH groups as compared to control groups, coinciding with low Bag3 expression. Here, we demonstrate for the first time that biphasic Bag3 expression (low in developing brain and increases with aging) is strongly inhibited by EtOH exposure. Bag3 is expressed at P5 and day P15 but not at day P8 showing the biphasic feature since there are 2 peaks. It was shown that Bag3 levels also fluctuate in mouse tissues and human cell lines. High or low Bag3 levels at certain timelines are probably due to developmental processes. Pivotal is an important developmental stage and Bag3 expression must be low at that period. These changes were accompanied by alterations in key signaling pathways in the brain, such as early postnatal accumulation of apoptotic proteins, developmentally regulated proteins, and neuronal loss.

### 3.2. Apoptosis in FASD.

We present novel data on the mechanism of neurotoxicity in human fetal brain. We show an association between EtOH, GA and apoptotic pathway alterations in fetal brain (Figure 3). We demonstrate that early EtOH exposure is linked to increases in neuronal injury as assessed by Caspase-3 activity. Maternal EtOH use in pregnancy is associated also with decreased synaptic protein expression in the fetal brain. This suggests that although controlled apoptosis is a critical neurodevelopmental process as human brain development progresses, but an imbalance and uncontrolled excess of apoptosis is problematic. Our results show that maternal use of EtOH is associated with increase of apoptosis and reductions in synaptic gene expression also in FB-Es. EtOH was associated with inhibition of autophagy proteins, and with increase of apoptotic protein levels.

### 3.3. Bag3 fluctuation.

Bag3 developmental expression was affected by EtOH. We provide behavioral observations at postnatal days of brain development from P5 to P8 and propose that Bag3 fluctuation period is important for pivoting. Two novel effects of maternal EtOH use on brain Bag3 content were observed: first, there was reduced Bag3 content in all EtOH-exposed brain samples; second, Bag3 expression was bi-phasic, with a peak expression at postnatal days P5 and P15, and with lowest levels of expression at postnatal day P8. This period was correlated with the pivoting behavior, suggesting the importance of Bag3 before and after P8, when pivoting was initiated, meanwhile, EtOH exposure could prevent this process by decreasing of Bag3. Another observation was that by inhibition of autophagy mechanisms, EtOH exposure could increase apoptotic events during development and movement.

### 3.4. Limitations.

In this study, we used fetal brain tissues and maternal blood from pregnant women who terminated their pregnancies. This approach presented some limitations. First, we need to increase the sample size for further studies. Next, it was impossible to do postnatal follow-up studies to correlate with the clinical outcomes. We partially circumvented the second limitation by correlating some of synaptic protein changes with the changes in eye diameter size, one of the important facial features in FASD. A larger follow-up study on FASD is currently in progress, where we collect blood and urine samples to verify EtOH use, and the children will be followed up to determine whether biomarkers found in our studies will predict an at-risk child with FASD.

## Conclusions

Here, we present an association between prenatal EtOH exposure and reduced markers of autophagy in the fetal brain in human and murine models. Inhibition of Bag3 may result in accumulation of GSK3β and its downstream targets, suggesting dysregulation of β-Catenin signaling. Prenatal EtOH exposure can affect early developmental changes in fetus and delay movement functions (pivotal) in neonates. Synaptic proteins were inhibited by EtOH during development in both, rat and human brains, which may reflect altered synaptic plasticity. Parallels between murine and human findings will help translating findings from animal models to humans to better understand human diseases. EtOH exposure is associated with reduced expression of synaptic proteins also in fetal brain-derived exosomes. Reduced levels of synaptic genes in FB-Es might serve as novel biomarkers to predict FASD in fetuses exposed to EtOH and might even point to possible therapies to prevent or ameliorate FASD.

## 4. Materials and Methods

### 4.1. Clinical recruitment.

First, and second trimester brain tissue and maternal serum were collected from women undergoing elective pregnancy termination under Temple University IRB approved protocols (**#21476:** Early Gestation Alcohol Exposure: Mechanisms of Human Developmental Injury, PI Dr. Darbinian, Nune). A face-to-face history was conducted by a trained study coordinator. The amount of EtOH was calculated as the total number of drinks consumed in a week multiplied by the number of weeks of exposure. A detailed questionnaire was used based on the NICHD PASS study. Each drink was estimated as the equivalent of one shot (1.5 oz of brandy or 5 oz of wine). Samples were collected between 9- and 23-weeks GA, as summarized in Table 1.

### 4.2. Cell Culture.

Human primary cortical neurons were prepared using a method developed by Dr. Darbinyan [22, 23]. In brief, after careful removal of the meninges, intact human fetal brain tissue (embryonic age 16 weeks approx 13g) was incubated with Tryple Express enzyme (Invitrogen, Carlsbad, CA) and DNase I (10 U/ml; Sigma, St. Louis, MO) at 37°C for 10–20 min, followed by three washes with Hibernate E medium. Tissue trituration was performed in culture medium (Neurobasal medium containing B27 supplement and 0.25 mM Glutamax) using a glass Pasteur pipette, and cells were plated on 60 mm poly-D-lysine-coated dishes (Sigma). Cytosine arabinoside Ara-C (Sigma), (final concentration 1 μM) was added after 16 hours for two days to reduce glial proliferation. Treatment of neuronal cultures with Ara-C (48 hours) efficiently depletes proliferating cells. At day 10 or 12 of in vitro culturing more than 98% of cells were positive for neuronal marker class III β-tubulin (verified by immunocytochemical analysis, [23]. Cells were maintained in Neurobasal medium supplemented with antibiotics (10 μg/ml gentamycin, 100 units/ml penicillin and 10 μg/ml streptomycin) and antifungal fungizone (Life Technologies, Inc.), 1 μg/ml, at 37°C in a humidified atmosphere containing 5% CO_2_.

### 4.3. Animal studies.

Detailed procedure on creating the rat FASD model was presented recently [10]. Female pregnant rats were maintained on an ethanol-containing (6.8% v/v) liquid diet for 6 days until delivery based on an approved procedure by Temple University IACUC protocol. Rats were then watched carefully until delivery and on days P2, P5, P8 and P15. Controls as well as ethanol-exposed pups were studied on their 2^nd^, 5^th^, 8^th^ and 15^th^ day. Pups were weighed, measured for crown rump length, and analyzed for ocular abnormalities. Eye size, length, and pupil size and shape were noted for both the right and left eyes of all fetuses and informative comparisons were made.

### 4.4. Treatment of neuronal cells.

Human cortical neuronal cells were incubated with EtOH (50 mM) for 48 h. The number of neurons and neuronal processes was measured in the presence of EtOH.

### 4.5. Isolation of Fetal Brain-Derived Exosomes (FB-Es) from Maternal Serum.

Human FB-Es were isolated as described previously [13, 14].

### 4.6. ELISA Quantification of Exosomal Proteins.

ELISA, a plate-based assay technique was used for detecting and quantifying synaptic proteins Synaptophysin, Neurogranin, Synaptotagmin and Synaptopodin, BDNF and CD81, used for normalization (American Research Products-Cusabio). Proteins were quantified according to the instructions. Levels of synaptic proteins were quantified by human ELISA kits for synaptophysin (American Research Products/CSB-E17406H, Waltham, MA, USA), synaptotagmin (BioMatik-EKU07545, Wilmington, DE, USA), synaptopodin (BioMatik-EKU07541, Wilmington, DE, USA), and neurogranin (American Research Products/CEA404HU, Waltham, MA, USA). ELISA data were statistically evaluated using Excel (Microsoft 365, software version 2404) and statistical analysis tools: CurveExpert for ELISA statistics (CUSABIO) or APP 96-well Plate Assay Data Analysis Software 5.0.apk (Cloud-Clone, Katy, TX, USA), available online.

### 4.7. Preparation of protein extracts from neuronal cells and immunoblot analysis.

Neuronal cells were incubated with EtOH for 48 hours. For preparation of whole-cell extract, cells were washed with cold phosphate-buffered saline (PBS) and solubilized in lysis buffer (50 mM Tris-HCl, pH 7.4, 150 mM NaCl, 0.1 % Nonidet P-40 and 1% protease inhibitors cocktail (Sigma). Cell debris was removed by centrifugation for 5 min at 4°C. For western-blot analysis, thirty micrograms of proteins were eluted with Laemmli sample buffer, heated at 95°C for 10 min, separated by 10% sodium dodecyl sulfate-polyacrylamide gel electrophoresis (SDS-PAGE), and transferred to supported nitrocellulose membranes (Bio-Rad) for 2 hours at 4°C, as described previously [18]. Proteins were visualized with the enhanced chemiluminescence detection system, ECL+ according to the manufacturer’s instructions (Amersham Pharmacia), and exposed to X-ray film. Western blot analysis of extracts from cells was performed for Bcl2, and the housekeeping protein, Grb2.

#### Preparation of total protein extracts from brain tissues and immunoblot analysis.

Homogenization, lysis, and western blotting were performed as previously described [25, 18]. The blots were subsequently washed three times and bound antibody detected either with the ECL kit or with the LI-COR system. For the LI-COR system, blots were incubated with IRDye^®^ 800CW Goat Anti-Rabbit and IRDye^®^ 680RD Goat Anti-Mouse Li-COR dyes and visualized with an Odyssey^®^ CLx Imaging System (LI-COR, Inc., Lincoln, NE) using Odyssey software (LI-COR Biosciences, Lincoln, NE, USA).

### 4.8. Synaptosome extract preparation.

Human fetal synaptic vesicles and cytoplasmic extracts were prepared from snap frozen brain tissue using Syn-Per synaptic Protein Extraction kit (Reagent #87793, Thermo Scientific). Synaptic vesicle proteins (50ug) were separated by gradient SDS-PAGE and transferred to nitrocellulose membrane. Proteins were detected using specific primary antibodies and secondary IRDye^®^ antibodies with the Odyssey^®^ CLx Imaging System.

### 4.9. Neurotoxicity/apoptosis (Caspase-3) assay.

Apoptotic Caspase-3/7 levels were assessed in brain and synaptic extracts using quantitative Real-Time RT-PCR and the Caspase-Glo 3/7Assay.

### 4.10. Caspase-GLO 3/7 activity assay.

Apoptosis was assessed by analysis of activation of caspase-3 using the substrate DEVD-aminoluciferin from the Caspase-Glo^™^ 3/7 assay kit (Promega, Madison, WI, USA), according to the manufacturer’s instruction. Approximately 1,000 of EtOH-treated cells, grown in a 24-well tissue culture plate, or brain cell lysate were analyzed in a final volume of 100 microliters culture medium per well. One hundred microliters of Glo reagent (1:1) were added to the culture medium and cell lysis was induced by shaking the cells for 2 min at room temperature. Subsequently, the supernatants of the different wells were transferred to the microcentrifuge tubes and the luminescent signal was stabilized for 10 min at room temperature. Luminescence was recorded as RLU/sec on a Femtomaster FB12 Luminometer (Zylux Corporation). Data were analyzed using Excel software. The histogram shows fold increase of Caspase 3/7 activity, and the error bars show the standard deviation from three independent readings.

### 4.11. Synaptic plasticity array.

The expression of a focused panel of 84 human genes associated with synaptic pathways (Qiagen) was analyzed and quantified by quantitative real-time RT-PCR.

### 4.12. Real-Time RT-PCR.

cDNA for q-PCR, was obtained using the total RNA isolated kit (Qiagen) and 1st strand RT synthesis (Qiagen), and Synaptogenesis Pathway Kit (Qiagen) according to the manufacture’s protocols. The expression of a focused panel of 84 human genes associated with synaptogenesis pathways was analyzed and quantified. StepOnePlus Real-Time PCR system thermo cycler was used. GAPDH and actin housekeeping genes were used for the normalization.

### 4.13. Droplet Digital PCR (ddPCR).

For absolute quantitation of mRNA copies, ddPCR was performed using the QX200 ddPCR system. Fifty nanograms of human fetal total RNA were used with the 1st Strand cDNA Synthesis Kit (Qiagen, Valencia, CA, USA). After reverse transcription, the cDNA (300 fold dilution) aliquots were added to the BioRad master mix to conduct ddPCR (EvaGreen ddPCR Supermix, BioRad, Hercules, CA, USA). The prepared ddPCR master mix for each sample (20-μL aliquots) was used for droplet formation. PCR conditions: Activation 95 °C 5 min, PCR 45 cycles at 95 °C 10 s, 60 °C 20 s, 72 °C 30 s, melting curve (95–65 °C), cool to 40 °C 30 s. The absolute quantity of DNA per sample (copies/μL) was calculated using QuantaSoft Analysis Pro Software (AP) (Bio-Rad, Hercules, CA, USA) to analyze ddPCR data for technical errors (Poisson errors). With 20,000 droplets, the above ddPCR protocol yields a linear dynamic range of detection between 1 and 100,000 target mRNA copies/μL. The estimated error is negligible compared with other error sources, e.g., pipetting, sample processing, and biological variation. The ddPCR data were exported to Microsoft Excel (Microsoft 365) for further statistical analysis.

### 4.14. Phospho-MAPK kinase array.

Human phospho-MAPK array kit was used (R&D Systems, Inc., Minneapolis, MN). Neuronal cells were lysed and used for phospho-MAPK arrays per the manufacturer’s protocol.

### 4.15. Methylthiazoletetrazolium (MTT) Assay.

For the MTT assay, we used a cell proliferation kit (MTT) according to the manufacturer’s protocol (Roche Applied Sciences, Indianapolis, IN USA). Neuronal cells were plated into 96-well plates in triplicate in two sets at a density of 10,000 cells/well and incubated with EtOH, at 50 mM/ml. After 48 hours, 10 μl MTT (5 mg/ml) were added to the wells (final concentration, 0.5 mg/ml) for 4 hours, and the reaction was stopped by the addition of 100 μl of solubilization solution. Viable cells with active mitochondria cleave the tetrazolium ring into a visible dark blue formazan reaction product, which was quantified by spectrophotometry in a Dynex MRX Revelation microplate reader (Dynex Technologies, Chantilly, VA) at 570 nm with a reference wavelength of 650 nm. The relative cell viability (percent) was determined as the ratio of average absorbance for treated cells to that for mock, untreated cells.

### 4.16. RNA Preparation and qRT-PCR.

Total RNA was isolated using the RNeasy kit (Qiagen, Valencia, CA) with on-column DNA digestion. The RT-PCR reaction was performed with 1 μg total RNA, using One-Step FAST RT-PCR Syber Green mix (Qiagen) on a Step One machine (Appled Biosystems). PCR conditions were activation 95°C 5 min, PCR 45 cycles: 95°C 10 sec, 60°C 20 sec, 72°C 30 sec, melting curve (95–65°C), cool to 40°C 30 sec. For relative quantification, the expression level of genes was normalized to the housekeeping gene β-actin. Results were presented in arbitrary units. The primers were used: β- actin, S 5′-CTACAATGAGCTGCGTGTGGC-3′, AS 5′-CAGGTCCAGACGCAGGATGGC-3′.

### 4.17. Gender Determination.

Gender determination was carried out using SuperScript One-Step RT-PCR with Platinum Taq (Life Technologies), and a BioRad C1000 Touch Thermal Cycler. In parallel studies, total cellular genomic DNA was isolated from fetal brain tissue for PCR analysis using the QIAamp DNA isolation kit (Qiagen). Amplification was performed in a GeneAmp PCR System 2400. Products were visualized by gel electrophoresis using a 2% agarose gel and GelRed DNA stain. The thermal cycler program used was: 45–55°C for 15–30 minutes, 94°C for 2 minutes, 55–60°C for 30 seconds, 68–72°C for 1 minute, 72°C for 5–10 min, and 12°C holding temperature. The Forward and Reverse gender determining primers used were (5’-CATGAACGCATTCATCGTGTGGTC-3’) and (5’-CTG CGG GAA GCA AAC TGC AAT CTT-3’) respectively.

### 4.18. Quantitative Western Blot Assays.

Changes in apoptotic or autophagy protein levels in rat brain were measured by quantitative western blotting, as previously described [18]. Loading dose was determined by protein concentration. Proteins (30 μg) in Laemmli sample buffer were heated at 95 °C for 10 minutes, separated by gradient SDS-PAGE and transferred to a NC membrane. Proteins were detected using specific primary antibodies (1:1,000 dilution) and secondary IRDye^®^ dyes (IRDye^®^ 800CW Goat Anti-Rabbit and IRDye^®^ 680RD Goat Anti-Mouse Li-COR dyes, 1:10,000) with the Odyssey^®^ CLx Imaging System (LI-COR, Inc., Lincoln, NE, USA). Band intensity (normalized to Grb2,) was detected, visualized and quantified using iS Image Studio^™^ Software version 3.1.

### 4.19. Antibodies.

Anti-α-tubulin clone B512 was purchased from Sigma-Aldrich (Sigma-Aldrich Co, St. Louis, MO). Neuronal class III β-tubulin (TUJ1) monoclonal antibody (Alexa Fluor-labeled, catalog No. A488–435L) was obtained from Covance (Berkeley, CA). Loading control mouse monoclonal Grb2 was obtained from BD Biosciences (San Jose, CA). Antibodies were purchased from EMD Millipore (Billerica, Massachusetts), from Sigma (St. Louis, MO), Aviva Systems Biology, Corp. (San-Diego, CA), OriGene (Rockville, MD).

### 4.20. Microscopy.

Fluorescence images of neuronal cells were visualized with an inverted Olympus fluorescence microscope using IPLAB software. Contrast and brightness were adjusted equally for all images using Adobe Photoshop version 5.5.

### 4.20. Statistical Analysis.

Statistical analysis was performed using SPSS Statistics from IBM Corp., released in 2017 for Windows, Version 25.0 (Armonk, NY, USA). All data are represented as the mean ± SD for all performed repetitions. Means were analyzed by a one-way ANOVA, with Bonferroni correction, where appropriate. Statistical significance was defined as p < 0.05. Sample numbers are indicated in the figure legends.

### 4.21. Ethics: Human Subjects.

Consenting mothers were enrolled at between 9 and 23 weeks’ gestation, under a protocol approved by our Institutional Review Board (IRB). This protocol involved no invasive procedures other than routine care. Maternal EtOH exposure was determined with a face-to-face questionnaire that also included questions regarding many types of drugs/medications used [18,26]. The questionnaire was adapted from that designed to identify and quantify maternal EtOH exposure in the NIH/NIAAA Prenatal Alcohol and SIDS and Stillbirth (PASS) study [27].

All procedures involving collection and processing of blood and brain tissues were done according to NIH Guidelines through a trained Study Coordinator. All investigators were trained annually to complete Citi Program - Human Subject training, Biohazard Waste Safety Training and Blood–Borne Pathogens Training, and all other required training. Written informed consent has been obtained from the patients for studies, and de-identified samples were used for this publication.

#### Eligibility Criteria.

The blood and placenta samples were obtained according to NIH Guidelines through a trained Study Coordinator. Samples were collected regardless of sex, ethnicity, and race. Subjects were excluded if they had an active urinary tract infection on history, nitrates or WBCs on clinical UA; no prisoners; no adults who are cognitively impaired or physically unable to provide consent to participate; no patients with severe blood disorders (e.g., hemophilia).

#### Treatment Plan.

Each patient was asked to sign a separate consent form for research on blood and tissue samples. Blood obtained was processed for collection of serum. No invasive procedures were performed on the mother, other than those used in her routine medical care. Placenta tissues were processed for protein isolation.

#### Risk and Benefits.

There were very small risks of loss of privacy as with any research study in which protected health information is viewed. The samples were depersonalized before they were sent to the lab for analysis. There were no additional risks of blood sampling as this was only performed in subjects with clinically indicated venous access. There was little anticipated risk from obtaining 2–3 cc of blood, but a well-trained Study Coordinator collected all samples.

There was no direct benefit to the research subjects from participation, but there is significant potential benefit for the future FASD subjects and the general population. This research represents a reasonable opportunity to further the understanding, prevention, or alleviation of a serious problem affecting the health or welfare of FASD patients.

#### Informed Consent.

Consent forms were maintained by the Study Coordinator and were not sent to the investigator with the samples. The de-identified log sheets and IRB protocol were sent by the Study Coordinator to the Principal Investigator with each blood and tissue sample.

## Figures and Tables

**Table 1:** Clinical characteristics of subjects used in the EtOH experiments.

Clinical characteristics of subjects	EtOH-consumers (n=20)	Controls (no EtOH, n=20)
Maternal Age (years ±SD)	25.43 ± 1.15	23.37 ± 2.41
Gestational Age (weeks ±SD)	15.33 ± 1.12	15.85 ± 1.57
Race: White vs Black (%)	50/50	50/50
Fetal Sex (male/female, %)	50/50	50/50

Maternal blood used in studies. A. Maternal blood samples from EtOH-exposed cases (n=20) and Controls (n=20) were matched individually for fetal sex, GA, and maternal age. PCR for SRY gene on Y chromosome for sex determination was performed for EtOH cases and control samples.

**Table 2.** Major human synaptic plasticity genes implicated in CNS development and disease.

Human Synaptic Plasticity PCR Array
The Human Synaptic Plasticity PCR Array profiles the expression of focused panel of 84 key genes central to synaptic alterations during learning and memory, involved in synaptic plasticity, LTP and LTD. The brain recalls immediate events via short-term memories; however, it must consolidate these events into long-term memory for later recall. Memory consolidation requires synaptic plasticity characterized by physical changes to, and gene expression changes in, neuronal synapses. Synaptic plasticity studies have discovered immediate-early genes (IEGs) that alter expression immediately after neuronal events. IEGs mediate long-term potentiation (LTP), a process that enhances synaptic connections and consolidates memories. However, as not all events become long-term memories, the opposite synaptic remodeling response, long-term depression (LTD), also plays a central role in synaptic plasticity Gene expression changes associated with LTD yield physical changes in the neuronal synapse that recycle receptors and either enhance or inhibit synaptic connections. This array includes IEGs and other genes important for LTP and LTD, as well as key neuronal receptor genes and genes important for synapse remodeling, focused panel of genes involved in synaptic plasticity, LTP and LTD with this array.
**Immediate-Early Response Genes (IEGs)**; ARC, BDNF, CEBPB, CEBPD, CREB1, CREM, EGR1, EGR2, EGR3, EGR4 FOS, HOMER1, JUN, JUNB, KLF1D, MMP9 (Gelatinase B), NFKB1, NFKBIB (TRIPS), NGF, NPTX2, NR4A1, NTF3, PCDH8, PIM1, PLAT (tPA), RELA, RGS2, RHEB, SRF, TNF.
**Late Response Genes:** INHBA, SYNPO.
**Long Term Potentiation (LTP):** ADCY1, ADCY8, BDNF, CAMK2A, CAMK2G, CDH2 (N-cadherin), CNR1, GABRA5, GNAI1, GRIA1, GRIA2, GRIN1, GRIN2A, GRIN2B, GRIN2C, GRIN2D, MAPK1, MMP9 (Gelatinase B), NTF4, NTRK2, PLCG1, PPP1CA, PPP1CC, PPP3CA, PRKCA, PRKCG, RAB3A, YWHAQ (14–3–3).
**Long Term Depression (LTD):** GNAI1, GRIA1, GRIA2 GRIA3, GRIA4, GRIP1 GRM1, GRM2, IGF1, MAPK1, NOS1. NGFR, PICK1, PLAT (tPA), PPP1CA, PPP1CC, PPP1R14A(CPI-17), PPP2CA, PPP3CA, PRKCA, PRKG1.
**Cell Adhesion:** ADAM10, CDH2 (N-cadherin), GRIN2A, GRIN2B, NCAM1, PCDH8, PPP2CA, RELN, TNF.
**Extracellular Matrix & Proteolytic Processing:** ADAM10, MMP9 (Gelatinase B), PLAT (tPA), RELN, TIMP1.
**CREB Cofactors:** AKT1, CAMK2G, GRIN1, GRIN2A, GRIN2B, GRIN2C, GRIN2D, MAPK1 (ERK2), PPP1CA, PPP1CC.
**Neuronal Receptors:** EPHB2, GABRA5, GRIA1, GRIA2, GRIA3, GRIA4, GRIN1, GRIN2A, GRIN2B, GRIN2C, GRIN2D, GRM1, GRM2, GRM3, GRM4, GRM5, GRM7, GRM8, NTRK2.
**Postsynaptic Density:** ADAM10, ARC, DLG4 (PSD95), GRIA1, GRIA3, GRIA4, GRIN1, GRIN2A, GRIN2B, GRIN2C, GRM1, GRM3, HOMER1, PICK1, SYNPO. **Others:** KIF17, SIRT1

**Human synaptic plasticity genes implicated in CNS development and disease and assayed in this study. Immediate-Early Response Genes (IEGs):** *ARC, BDNF, CEBPB, JUN, JUNB, MMP9, NFKB1, NGF, PCDH8, TNF.* **Late Response in Synaptic Plasticity:** *SYNPO*. **Genes involved in Long Term Potentiation (LTP):** *BDNF*, *GRIN2C*, *MAPK1* (*ERK2*); **Long Term Depression (LTD):** *IGF1*, *MAPK1*; **Cell Adhesion Molecules:** PCDH8, *TNF;* **Extracellular Matrix (ECM) Molecules:** *MMP9;* **CREB Cofactors:** *AKT1*, *GRIN2C*, *MAPK1* (*ERK2*); **Neuronal Receptors:** *GRIN2C*; **and Other Synaptic Plasticity Genes:** *SIRT1*.

**Table 3:** Differential Effects of EtOH on the Expression of Synaptic Markers.

	Down-regulation	Up-regulation
**Synaptic markers (FB-E)**		
Synaptophysin	**↓ 5.6**	
Synapsin	**↓ 4.5**	
Synaptoptagmin	**↓ 1.7**	
Synaptopodin	**↓ 2.1**	
Neurogranin	**↓ 5.3**	
Bcl-XL	**↓ 3.2**	
Shh	**↓ 1.57**	
BDNF	**↓ 2.3**	
**Upstream to Synaptic Plasticity genes**		
miR-9	**↓ 2.2**	
REST	**↓ 3.6**	
HSF1	**↓ 2.1**	
β-catenin		**↑ 1.8**
**Synaptic plasticity (synaptosomes):**		
Synaptophysin	**↓ 5.1**	
Synapsin	**↓ 4.5**	
Synaptoptagmin	**↓ 1.3**	
Synaptopodin	**↓ 2.2**	
Neurogranin	**↓ 1.2**	
Active Caspase-3		**↑ 2.4**
**Synaptic plasticity (brain):**		
**Immediate-Early Response Genes (IEGs)**		
Arc		**↑ 1.2**
BDNF	**↓ 2.2**	
CEBPb	↓ **1.9**	
JUN	**↓ 2.1**	
JUNB	**↓ 2.4**	
p-JNK2	**↓ 9.8**	
MMP9		**↑1.9**
NFKB1	**↓ 2.8**	
NGF	**↓ 2.35**	
PCDH8	**↓ 1.22**	
TNF		**↑ 7.3**
**Late Response in Synaptic Plasticity**		
SYNPO	**↓ 1.3**	
**Long Term Potentiation (LTP)**		
BDNF	**↓ 2.2**	
GRIN2C	**↓ 1.32**	
MAPK1 (ERK2)	**↓ 1.9**	
**Long Term Depression (LTD)**		
IGF1	**↓ 4.9**	
MAPK1 (ERK2)	**↓ 1.9**	
**Cell Adhesion Molecules:**		
PCDH8	**↓ 1.22**	
TNF		**↑ 7.3**
**Extracellular Matrix (ECM) Molecules**		
MMP9		**↑ 1.9**
**CREB Cofactors**		
AKT1	**↓ 1.47**	
GRIN2C	**↓ 1.32**	
MAPK1 (ERK2)	**↓ 1.9**	
**Neuronal Receptors**		
GRIN2C	**↓ 1.32**	
**Other Synaptic Plasticity Genes**		
SIRT1	**↓ 1.28**	
**Neuronal markers**		
α-III Tubulin	**↓ 1.9**	
NF-L	**↓ 1.2**	
**Cytokines / Chemokines**		
TNF-β		**↑ 7.3**
IGF-1	**↓ 4.9**	
**Murine FASD (rat brain)**		
Bcl2	**↓ 1.7**	
p-GSK3β		**↑ 2.7**
β-catenin		**↑ 1.4**
Bag3	**↓ 8.2**	
Synaptophysin	**↓ 2.1**	
Active Caspase-3		**↑ 1.9**
**Apoptosis**		
Active Caspase-3		**↑ 1.9**
Bax		**↑ 2.3**
**Anti-apoptotic**		
Bcl-2	**↓ 1.7**	
Bcl-XL	**↓ 2.1**	
**Autophagy**		
Bag3	**↓ 8.2**	
LC3	**↓ 1.5**	

EtOH exposure and expression of synaptic plasticity markers. Markers of synaptic plasticity, apoptosis and autophagy. Levels of mRNAs and proteins for each group of synaptic plasticity were assayed in whole brain homogenates, and FB-Es, determined by qRT-PCR, ddPCR, ELISA and western-blot assay, and graphed as Fold Regulation. Values are normalized relative to controls (n = 20 per group, GA=9 to 23 weeks for Control and EtOH-exposed group). Student’s t-test; p-values based on parametric, unpaired, two-sample equal variance, 2-tailed distribution. EtOH exposure increases cytotoxic cytokines/chemokines expression and apoptotic proteins in fetal brain and inhibits survival and autophagy proteins. Effects of maternal EtOH use on expression of cytokines/chemokines in fetal brain during development. Cytokine transcription was assayed by Real-Time qRT-PCR for: TNF-α and IGF-1 mRNA from human fetal and rat pups brain. In animal studies (n = 16 pups total; 4 per each age group, the EtOH-exposed group, and control group; shown as fold expression relative to unexposed controls). Rat Brain: Caspase-3 and Bax were increased in the brain of EtOH pups (↑1.9 and ↑2.3), while the early expression of anti-apoptotic Bcl2 and biphasic Bag3 was inhibited by EtOH (1.7 and 8.2 folds). Phosphorylation of glycogen synthase kinase 3β (GSK3β) was increased with EtOH exposure. During the period of Bag3 inhibition, the level of a GSK3β downstream target, β-Catenin, was also increased in the brain of EtOH pups. Behavioral observations at postnatal days P8 and P15 revealed pivoting difficulties in the ethanol group when compared to controls, coinciding with low Bag3 expression, and reduced synaptophysin. Human FB-E: reduced expression of synaptic markers, synaptophysin and synapsin. Compared with controls, subjects with EtOH exposure showed abnormalities of synaptic signaling in the fetal brain and FB-Es. Decreased fetal neural exosome levels of neuronal survival factors in EtOH group with alcohol exposure (EtOH, *n* = 20). The significant differences were between levels of HSF1 (*p* < 0.05), Bcl-XL (*p* < 0.01), and REST (*p* < 0.0001) for the control and EtOH groups. Synaptophysin, synaptotagmin, synaptopodin, and neurogranin were significantly reduced in EtOH group (p <0.001 for all). Up to five-fold inhibition in microRNA-9 was observed in FB-Es from EtOH exposed groups compared with controls. Human fetal brain: In most synaptic plasticity pathways, levels of mRNAs were reduced (but ARC, MMP9 and TNF). **Immediate-Early Response Genes (IEGs):** *ARC* (↑120%), ↓*BDNF*, ↓*CEBPB*, ↓*JUN*, ↓*JUNB*, ↑*MMP9*, ↓*NFKB1*, ↓*NGF* (80%), ↓*PCDH8* (82%), ↑*TNF*. **Late Response in Synaptic Plasticity:** ↓*SYNPO* (78%). **Genes involved in Long Term Potentiation (LTP):** ↓*BDNF*, ↓*GRIN2C* (76%), ↓*MAPK1* (*ERK2*); **Long Term Depression (LTD):** ↓*IGF1*, ↓*MAPK1* (*ERK2*); **Cell Adhesion Molecules:** ↓PCDH8, ↑*TNF;* **Extracellular Matrix (ECM) Molecules:** ↑*MMP9;* CREB Cofactors: ↓*AKT1*, ↓*GRIN2C* (76%), ↓*MAPK1* (*ERK2*); **Neuronal Receptors:** ↓*GRIN2C* (78%); **Other Synaptic Plasticity Genes:** ↓*SIRT1*. Upregulated in EtOH cases, including the neurotoxic *TNF⍺*, immediate early gene *ARC*, which is trafficked to dendrites, and matrix metalloproteinase-9 (*MMP9)*.

## Data Availability

This study collected demographic, behavioral, and laboratory data from normal, healthy women and from women who drank alcohol during pregnancy. Our research team supports all these activities and has developed a data-sharing plan. We also recognize that additional benefits from data sharing may arise in the future that are not apparent at this time, and we are prepared to work specifically with NIH in addressing all requests for raw data. At the present time, we have not deposited any of these raw data in an existing databank, but will make the data available to other investigators on request, in a manner consistent with NIH guidelines. Consistent with NIH policy, shared data will be rendered “free of identifiers that would permit linkages to individual research participants and variables that could lead to deductive disclosure of the identity of individual subjects” Intellectual property and data generated under this project will be administered in accordance with both University and NIH policies, including the NIH Data Sharing Policy and Implementation Guidance of 5 March 2003, and 0925–0001 and 0925–0002 (Rev 07/2022 through 01/31/2026). With this caveat observed, data will be made available to the NIH/NICHD/NIAAA. Sufficient identifiers will be provided to the NIH so that research participants can be assigned a Global Unique Identifier (GUID), which is a universal subject ID that protects personally identifiable information (PII). Using the GUID, NDAR can bring together multiple types of data collected from a single participant, regardless of where and when those data were collected. Biological samples (blood, serum, exosomes, and RNAs) and data that are shared will be completely free of identifiers that would permit linkages to individual research participants. We will make biological samples, deidentified data, and associated documentation available to users only under a data-sharing agreement that provides for (1) a commitment to using the data only for research purposes, (2) a commitment to securing the data using appropriate computer technology; and (3) a commitment to destroying or returning remaining samples after analyses are completed. Intellectual property and data generated under this project will be administered in accordance with both University and NIH policies, including the NIH Data Sharing Policy and Implementation Guidance of 5 March 2003. As the FAIR data bank receives approval from the NIH, the data will be made available to that group as well. The NIH implemented a new policy for Data Management and Sharing, effective on January 25, 2023 (https://grants.nih.gov/grants/guide/notice-files/NOT-OD-21-014.html). We will adopt that policy also. Data will be also available at https://www.mdpi.com/ethics accessed on 1 January 2025.

## Abbreviations:

Bag3: Bcl2-associated athanogene-3
Bax: B-cell lymphoma-2 (Bcl2)-associating-X protein
Bcl-XL: B-cell lymphoma-extra large
ddPCR: digital droplet polymerase chain reaction
ELISA: enzyme-linked immunosorbent assay
EtOH: ethanol, alcohol
FAS: fetal alcohol syndrome
FASD: fetal alcohol spectrum disorder
FB-Es: fetal brain exosomes
HSF1: type 1 heat-shock factor
IEGs: Immediate-Early Response Genes
GA: gestation age
GSK3β: glycogen synthase kinase 3β
MMP9: matrix metalloproteinase-9
NC: nitro-cellulose membranes
PAGE: polyacrylamide gel electrophoresis
PAE: prenatal alcohol exposure
qRT-PCR: quantitative reverse transcription polymerase chain reaction
REST: restriction element-1 silencing transcription factor
SRY: sex-determining region of the Y chromosome
